# Noradrenergic therapies in neurodegenerative disease: from symptomatic to disease modifying therapy?

**DOI:** 10.1093/braincomms/fcaf310

**Published:** 2025-08-25

**Authors:** Robert Durcan, Claire O’Callaghan, James B Rowe

**Affiliations:** Department of Clinical Neurosciences and Cambridge University Hospitals NHS Foundation Trust, University of Cambridge, Cambridge CB2 0SZ, UK; Brain and Mind Centre and School of Medical Sciences, Faculty of Medicine and Health, University of Sydney, Sydney, NSW 2050, Australia; Department of Clinical Neurosciences and Cambridge University Hospitals NHS Foundation Trust, University of Cambridge, Cambridge CB2 0SZ, UK; MRC Cognition and Brain Sciences Unit, University of Cambridge, Cambridge CB2 7EF, UK

**Keywords:** noradrenaline, neurodegeneration, locus coeruleus

## Abstract

A feature shared by many different neurodegenerative diseases is early pathology and degeneration of the pontine locus coeruleus. The human locus coeruleus contains about 50 000 neurons and is the primary source of the neurotransmitter noradrenaline. We propose the hypothesis that noradrenergic drugs can have broad, transdiagnostic benefit in slowing or preventing the progression of neurodegenerative diseases. There are direct noradrenergic anti-inflammatory effects *in vivo*, with microglia and astrocytes regulated by adrenoreceptors, and noradrenergic influences on glymphatics. Noradrenaline loss is associated with a pro-inflammatory state, promoting further neurodegeneration. Noradrenergic neuron loss is associated with worsening of both amyloid and tau deposition in animal models. There may be indirect survival benefits arising from alleviating the prognostically detrimental features of apathy and impulsivity, and noradrenergic influences on other neurotransmitters. The evidence base we set out supports the need for clinical trials of noradrenergic treatments for disease-modification.

## Teaser

Durcan *et al*. propose that degeneration of the locus coeruleus and loss of noradrenaline contribute to the pathologic progression of neurodegenerative diseases. Noradrenergic drugs may offer broad benefits by reducing inflammation, improving protein clearance, while also alleviating symptoms. Clinical trials are warranted to test noradrenergic therapies as potential disease-modifying treatments.

## Graphical Abstract

**Graphical Abstract:**
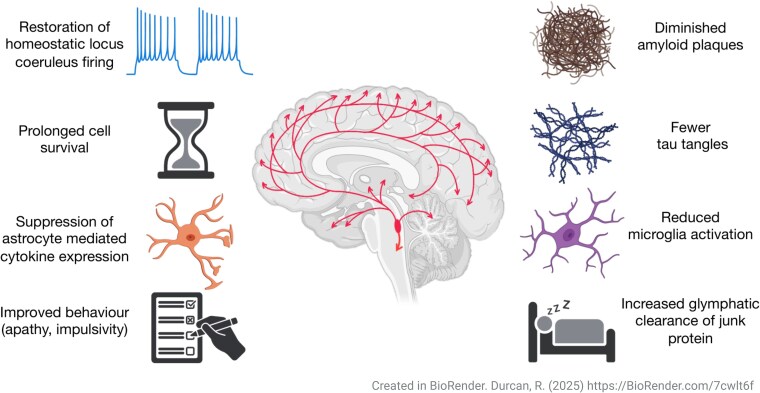

## Introduction

Neurodegenerative diseases remain a devastating group of illnesses, with high personal, societal and economic costs arising from dementia, Parkinsonism and autonomic impairment. As our societies age and survive longer, the number of people affected will rise rapidly.^1^ Although the common neurodegenerative diseases differ in their genetics, molecular pathology and syndromic characteristics, they also have important commonalities. One of the features shared by many different neurodegenerative diseases is early degeneration of the locus coeruleus noradrenergic system. This occurs in Alzheimer's disease,^2,3^ Parkinson's disease,^4^ progressive supranuclear palsy,^5^ corticobasal syndrome,^6^ multiple systems atrophy,^7^ frontotemporal dementia^8^ and dementia with Lewy bodies.^9^

Effective disease-modifying therapies have been elusive, despite progress in understanding the neurobiological targets and drugs that have been effective at acting on those targets in preclinical models. Notwithstanding some recent progress in anti-amyloid therapy (e.g. lecanemab and donanemab) for Alzheimer's disease,^10,11^ there remains a pressing need for clinically effective treatments. Currently licensed treatments are symptomatic, acting via pharmacological restoration of depleted neurotransmitters such as acetylcholine^12^ or dopamine.^13^ Here we focus on the noradrenergic system. This is not only because of its widespread role in cognitive functions, and emerging evidence of cognitive and behavioural benefits from noradrenergic treatments,^14^ but also because of the potential for modification of underlying disease pathobiology. There are already several, well-tolerated noradrenergic agents on the market, the repurposing of which might offer a cost-effective addition to management options of neurodegenerative disorders.

**Figure 1:**
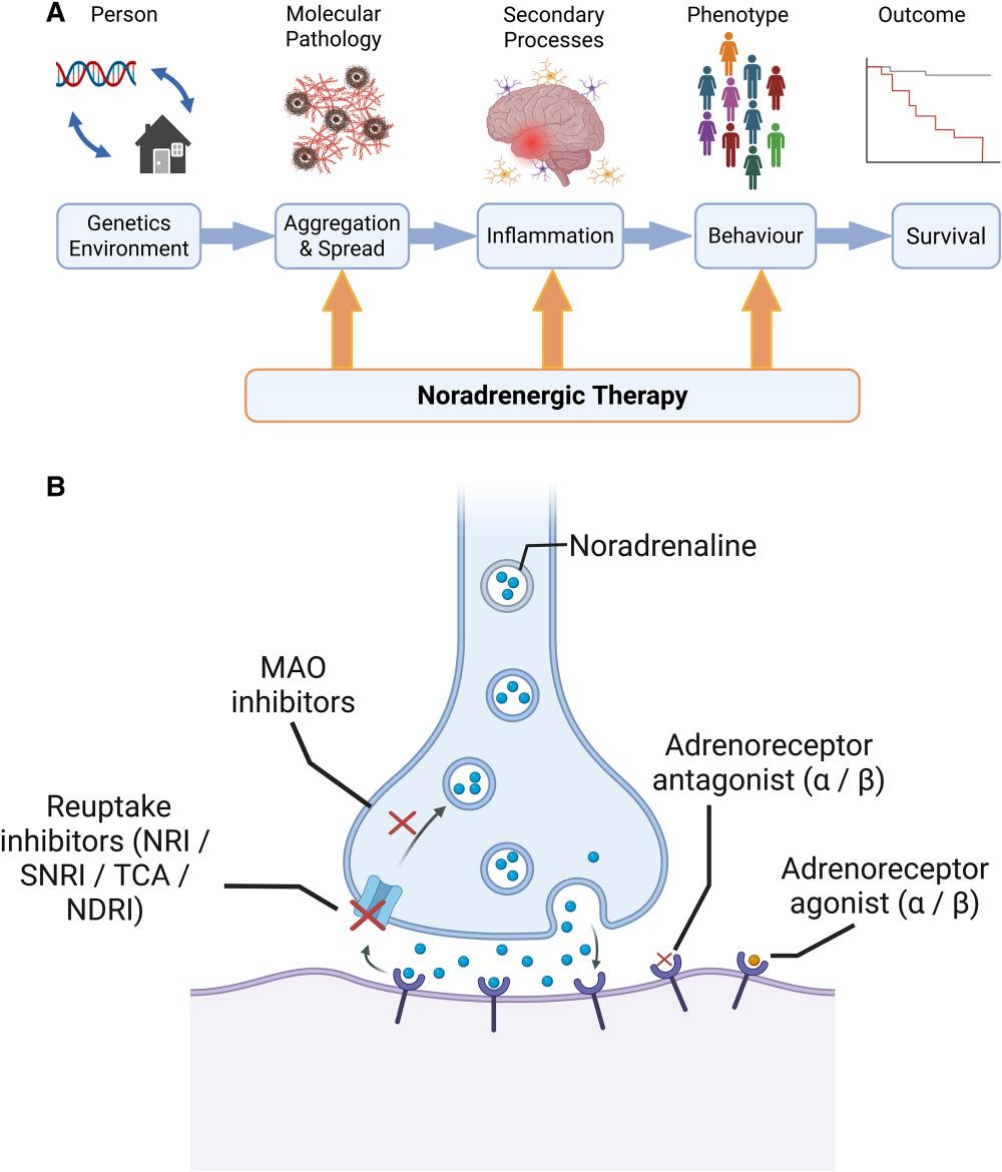
**Action of noradrenergic drugs: (A) Potential disease-modifying actions of noradrenergic therapy may arise at multiple points in the pathogenesis of neurodegenerative diseases and their consequences.** (**B**) There are multiple sites of action of noradrenergic treatments. NRI = Noradrenaline reuptake inhibitor, SNRI = Serotonin / Noradrenaline reuptake inhibitor, TCA = Tricyclic antidepressant, NDRI = Noradrenaline / Dopamine reuptake inhibitor. Created in BioRender. Durcan, R. (2025) https://BioRender.com/j93pbpn.

We propose that noradrenergic treatments have potential for both disease modification and symptomatic therapy. Treatments focussing on restoration of the diseased noradrenergic system may alter the course of neurodegenerative illnesses at multiple stages (Fig. 1). There are two fundamental reasons for this claim. First, noradrenergic drugs have *direct* effects *in vivo*, with anti-inflammatory properties which may be beneficial in slowing or preventing the degenerative cascade within and across this heterogeneous group of neurodegenerative diseases. Second, there may be *indirect* survival benefits that arise from alleviating the prognostically detrimental neuropsychiatric features of apathy and impulsivity. Indirect effects may also be exerted by noradrenergic influences on other neurotransmitter systems. We consider each of these mechanisms below, after summarising the evidence of noradrenergic deficits in diverse neurodegenerative diseases.

### Noradrenaline deficits are common and early in many neurodegenerative diseases

The locus coeruleus (LC) is a bilateral, cylindrical nucleus adjacent to the floor of the fourth ventricle, with only around 50 000 neurons in humans.^15^ It is the brain's primary source of the neurotransmitter noradrenaline.^16,17^ The LC noradrenergic system supports many cognitive functions, including vigilance, attention, cognitive control and memory.^18-20^ It is also a key regulator of autonomic function,^21^ circadian rhythm,^22,23^ pain modulation^24^ and neuroinflammation.^25^

**Figure 2:**
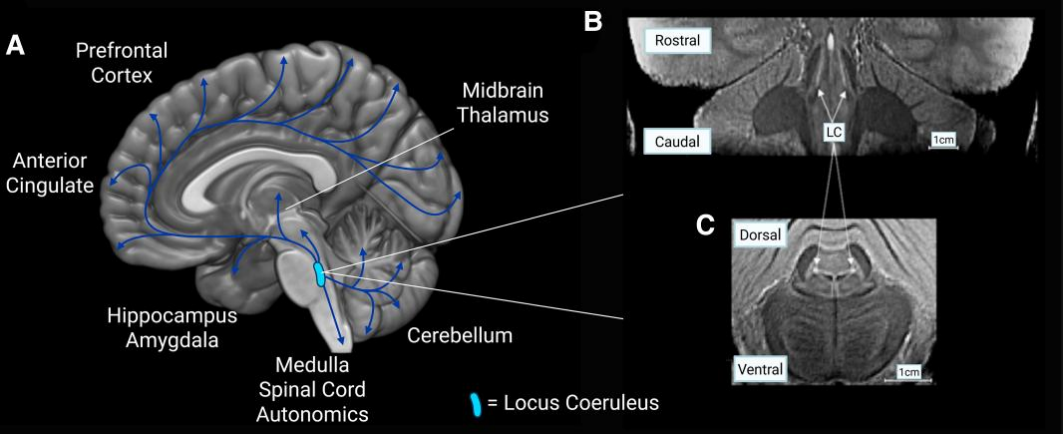
**Locus coeruleus projections: (A): Sagittal view of the brain, illustrating the widespread cortical projections from the locus coeruleus (LC), (B, C) Magnetic resonance images using a magnetisation transfer sequence, showing coronal and axial views of locus coeruleus respectively.** Created in BioRender. Durcan, R. (2025) https://BioRender.com/6q5x99s.

The locus coeruleus has widespread connections to the cortex and subcortical structures, with a coarse topographical organization (Fig. 2).^21,26^ The caudal locus coeruleus projects mainly to the brainstem, spinal cord, cerebellum and occipital cortex, while rostral locus coeruleus neurons project predominantly to the hippocampus, olfactory, frontal and parietal cortex.^27^ Early work suggested that the majority of afferent inputs into the LC arise from the medulla.^28^ However, it has since been shown that the LC receives broad inputs from midbrain, medullary, cerebellar and motor and prefrontal cortex neurons, and these same LC neurons have anatomically distinct afferent inputs depending on their efferent projections. For example, medullary projecting outputs receive less input from amygdala neurons than afferent neurons projecting elsewhere. This anatomical selectivity of inputs may enable the LC to modulate specific targets.^29^

LC activity is in constant flux, adapting dynamically to attentional demands and states of cognitive arousal. Tasks that are cognitively challenging are associated with acute ‘phasic’ locus coeruleus firing. This occurs on a background of a baseline ‘tonic’ firing. The dynamic interplay between tonic and phasic modes of LC activity plays a critical role in optimizing cognitive performance, with phasic bursts enhancing the signal-to-noise ratio in neural processing during tasks that require focused attention and mental effort.^30-32^

A long pre-symptomatic prodrome with proven neuropathological change is seen across the spectrum of neurodegenerative disorders, irrespective of the underlying aetiopathogenesis, including β-amyloid, α-synuclein, phosphorylated tau and TDP-43.^2,33-37^ Neuropathological inclusions, cell death and atrophy of the locus coeruleus occur early in many neurodegenerative diseases.^25,38^ This predates cortical involvement in Alzheimer's disease.^2,3^ There is early involvement with neurofibrillary tau in the LC and later emergence of cell loss.^39^ Post-mortem work demonstrates that from early stages in AD (Braak stage 0–2), there is reduced LC volume, and stereology indicates LC cell loss from Braak stage 3 onwards.^40^ It is unclear if this sequence is a time effect or requires an as-yet-undefined secondary trigger. Structural MRI evidence of involvement of the locus coeruleus in asymptomatic, amyloid-β negative individuals is associated with a substantially increased risk of becoming mildly cognitively impaired, implying that the locus coeruleus may offer a radiological biomarker of early Alzheimer's disease,^41^ although the biological explanation for the emergence of cognitive symptoms in this setting remains uncertain. A combination of *in vivo* and post-mortem work indicates that early pathology of the locus coeruleus is also seen in Parkinson's disease,^4^ progressive supranuclear palsy,^5^ corticobasal degeneration,^6^ multiple systems atrophy^7^ and dementia with Lewy bodies.^9,42^ An apparent exception with conflicting data is the behavioural variant of frontotemporal dementia (bvFTD),^43,44^ possibly reflecting heterogeneity of its pathogenesis. There is a lower LC signal on MR (interpreted as greater locus coeruleus degeneration) in early-onset Alzheimer's Disease than in the more commonly observed late-onset Alzheimer's disease, suggesting a potential biologically selective vulnerability in the younger cohort.^45^ Post-mortem analysis suggests that locus coeruleus pathology is more severe in tau-associated disorders versus those with predominant TDP-43 involvement, which may explain the mixed reports in bvFTD.^8,46^ The hypotheses remain to be tested that symptoms associated with locus coeruleus loss (e.g. apathy) are predictive of underlying tau pathology in bvFTD, particularly a 4-repeat tau pathology, versus 3-repeat tau pathology.^47^

There is no uniform pattern or distribution of locus coeruleus pathology across these neurodegenerative conditions. *Post-mortem* data support a heterogeneous pattern of degeneration of the locus coeruleus, with Alzheimer's disease affecting predominantly the rostral locus coeruleus, while Parkinson's disease affects the caudal locus coeruleus earlier and more severely.^39,48^ This may influence disease phenotypes, given the topography of locus coeruleus projections and the functions of target regions. A noradrenergic phenotype has been proposed for Parkinson's disease, with dominant non-motor features at the outset. These non-motor features of Parkinson's disease are conventionally poorly responsive to dopaminergic medication.^49^ Indeed, atrophy of noradrenergic neurons in the locus coeruleus occurs at a faster rate than the better-known dopaminergic loss in the substantia nigra in Parkinson's disease.^50^ Patients with Parkinson's disease who displayed prominent noradrenergic (non-motor) phenotypes at diagnosis have been shown to have faster disease progression, offering up a hypothesis that the associated noradrenaline deficit may be associated with accelerated neurodegeneration.^51,52^

*In vivo* imaging has shown a non-linear association between advancing age and LC contrast, using LC-sensitive sequences.^53,54^ An age-related plateau, or even decline, with age is reported particularly for the rostral LC regions.^55^  *Post-mortem* studies have suggested age-related atrophy of the noradrenergic system in older adults, but this is less severe than in symptomatic neurodegenerative disease.^31^ Indeed, stereological quantitative methods suggest that age-related atrophy of the locus coeruleus is minimal in the absence of Alzheimer's pathology elsewhere in the brain.^40^  *In-*vivo imaging incorporating Aβ and tau PET supports that this age-related change in LC contrast in rostral regions is driven by individual accumulation of latent (non-symptomatic) AD pathology.^56^ Given the common prevalence of early AD pathology with age, studies showing a relationship between age, cognition and LC integrity may have been influenced by early AD changes.^57^ Correlation has been drawn between locus coeruleus integrity in cognitively asymptomatic patients and subsequent clinical Alzheimer's disease diagnosis within two years.^58^ It remains to be seen if this locus coeruleus integrity loss is merely an early biomarker of sub-clinical disease or a possible contributory mechanism to disease progression

## Measuring the noradrenergic system

While the LC does not display sufficient contrast to be readily visualised by conventional clinical MRI sequences, modern imaging techniques allow *in vivo* quantification of the noradrenergic system. Drawing upon pathological evidence that noradrenergic cells are rich in neuromelanin, magnetisation transfer MRI sequences have been developed that display contrast that is anatomically consistent with the LC.^59^ This LC signal has been pathologically correlated with noradrenergic neurons at *post-mortem.*^60^ It is unclear if this signal is exclusively driven by neuromelanin within noradrenergic neurons, given the neuromelanin-free contrast in other species and the high water content of LC noradrenergic neurons.^61,62^ The biologic basis of this LC imaging signal does not directly affect the potential therapeutic effects of treatments targeting the noradrenergic system. The MT-sensitive LC signal increases in early adult life, to about 50 years, before plateauing. The age-related effect was stronger in males than in females in one study.^63^ Reductions in this LC signal have been seen to varying degrees across the spectrum of neurodegenerative diseases. In vivo imaging studies demonstrate a coarse disease-specific spatial pattern, whereby LC signal loss is greatest in rostral regions in AD, and in caudal regions in PD and other Parkinsonian syndromes.^23,64-70^ This imaging work is supported by the differing rostral–caudal patterns of pathological involvement seen in Alzheimer's^71^ and Parkinson's diseases^64,65,72^ at post-mortem. Although several studies show a relationship between mild cognitive impairment and LC signal,^73-76^ this is not a universal finding.^77^ However, even in the latter study showing no imaging correlate, LC integrity correlated with CSF levels of Aβ.^77^ Longitudinal imaging studies of the LC and noradrenergic system are scarce, but Betts *et al.* showed that the degree of interval loss of LC signal correlates with poorer cognitive outcomes.^78^

PET Ligands have been developed that selectively bind to the noradrenaline transporter.^79^ Work with these ligands has provided further supportive evidence of the early involvement of the locus coeruleus in neurodegenerative disease.^80,81^ A helpful addition of PET imaging over the structural magnetisation-transfer (MT) MRI protocol is that it enables quantification of the cortical uptake of noradrenaline. [18F]Fluoro-m-tyrosine, a less selective ligand that quantifies catecholamine synthesis, has been studied, showing potential interactions between functional capacity of the noradrenergic system, LC structural integrity as measured with MR and cognitive function.^82^

Resting-state functional MRI has been used to assess locus coeruleus connectivity in young healthy controls, with promising results.^83,84^ Coupled with tractography and diffusor tensor imaging, fMRI has shown that the LC and other brain regions that have high functional connectivity also have high structural connectivity.^83^ Alterations in patterns of locus coeruleus thalamocortical connectivity have been seen in mild cognitive impairment,^85^ and in Alzheimer's disease with depression.^86^ In an ageing noradrenergic system, there is compensatory upregulated activation of the LC to emotionally salient stimuli, further complicating the interpretation of functional imaging data.^87^ A limitation of fMRI in studying the LC is that conventional voxel sizes used at 3T (typically 2–3 mm, plus smoothing) are bigger than the LC width, and extra-LC signals may contribute to the BOLD signal.^88,89^ The small number of neurons in LC, the proximity to the fourth ventricle with physiological noise from CSF, and pulse or respiration artefacts, increase the challenge of LC fMRI.^90^

*In vivo* quantification of noradrenergic system activity is also possible by analysing metabolites in cerebrospinal fluid. Measurement of mono-amines in cerebrospinal fluid is technically challenging, but a trend towards lower noradrenaline levels in Alzheimer's disease *versus* controls has been reported.^91,92^ Cortical pathology may also affect the metabolism of noradrenaline, making interpretation of cerebrospinal fluid assays more difficult. Another factor making interpretation of noradrenaline metabolite measurement difficult is that, in addition to the conventional synaptic neurotransmission of noradrenaline, it has extra-synaptic modulatory influences, known as volumetric transmission.^93^ Therefore, a similar absolute quantity of noradrenaline may have very different clinical effects, depending on the integrity of noradrenaline receptors. Measurement of metabolites in blood is not reflective of what is happening in the brain, as noradrenaline has poor permeability across the blood–brain barrier.^92^

Pupillometry has been used for the *in vivo* assessment of the noradrenergic system, with pupil dilatation providing an indirect measure of phasic firing of the locus coeruleus in humans,^94-96^ rodents^97^ and primates.^98^ The LC displays baseline low-level ‘tonic’ firing, with super-imposed faster ‘phasic’ firing bursts, which are attention-mediated.^30-32^ Resting pupil diameter is proposed to reflect the intrinsic responsivity to the locus coeruleus noradrenergic system.^30-32,94,98^ Evidence from fMRI suggests that locus coeruleus dysfunction may manifest as impaired task-related phasic pupil responses, occurring on a background of elevated tonic noradrenaline levels as indicated by higher BOLD signal variance in the ventral attentional network.^98,99^ These pupil dilatation responses may be a useful, non-invasive proxy of noradrenergic treatments as a measure of target engagement and/or drug response in clinical trials. For example, an increase in peak amplitude or velocity of pupil dilatation while ‘on-drug’ may suggest improved phasic firing. Conversely, baseline ‘resting’ pupil diameter may change with restored noradrenergic functioning and higher baseline tonic firing. Both baseline increases and performance-related phasic increases in pupil diameter in people with Parkinson's disease followed a single dose of the noradrenergic reuptake inhibitor atomoxetine.^100^ Pupillometry may offer an opportunity to observe changes in phasic-to-tonic locus coeruleus firing patterns, which are thought to characterise early neurodegenerative pathologies.^101^ Pupil size is only a proxy measure, with modulating factors that include age,^102,103^ serotonergic innervation from the raphe nucleus,^104^ parasympathetic innervation of the pupillary sphincter muscle,^105^ extra-striatal off-target effects of dopamine,^106^ and potential indirect effects of orexin^107^ and histamine.^108^ Relevant to neurodegenerative disease, both resting (tonic) pupillary diameter, and amplitude and velocity of pupillary light reflex (phasic) are lower in AD versus controls.^109^

## Noradrenergic medication as a symptomatic treatment

Given that a) noradrenaline is important for normal cognition, and b) noradrenaline is commonly depleted in neurodegenerative illnesses, it is a testable hypothesis that restoring function of the noradrenergic system provides symptomatic benefit in neurodegenerative diseases (Table 1). This psychopharmacological benefit of noradrenergic treatment on cognition and behaviour has been established across heterogeneous patient groups with neurodegenerative diseases.^111-115,133,151,152^

**Table 1:** Common noradrenergic treatments and the evidence to support their use in neurodegenerative diseases

Drug Name	Indications/Common Use	Mechanism of Action	Other Systems Affected	Studied in Neurodegeneration?
Atomoxetine	ADHD	Selective NA reuptake inhibitor (NRI)	Increases extracellular DA levels in regions outside the striatum.^110^ Minimal serotonin effect	AD: 2 × Phase II RCT—No cognitive benefit^111^ but reduced CSF Tau, pTau and biomarkers of synaptic health^112^. PD: No effect on depressive symptoms^113^. Mixed reports of effect on overall cognition^113,114^ Improved measures of executive dysfunction^115^
Reboxetine	Depression	NA reuptake inhibitor	Inhibits DA reuptake at the NA transporter, but much weaker than NA inhibition^116^	PD: Non-randomised trial: Improvement in observer rated depression and anhedonia at 4 weeks^117^
Desipramine	Depression, neuropathic pain	TCA; inhibits NA > serotonin reuptake	Weak serotonergic and anticholinergic affects	AD: Improved functional independence versus placebo.^118^ PD: Outperforms placebo and citalopram for depressive symptoms^119^
Nortriptyline	Depression, neuropathic pain	TCA; inhibits NA reuptake > serotonin	Weak serotonergic and moderate anticholinergic effects	PD: Benefit in treating depressive symptoms, versus placebo and SSRI (paroxetine).^120^ PSP: Case report of benefit on depressive and motor symptoms^121^
Amitriptyline	Depression, neuropathic pain, migraine	TCA; inhibits serotonin reuptake > NA	Strong serotonergic and anticholinergic effects	PSP: Case report of benefit for motor symptoms^122^
Venlafaxine	Depression, anxiety	SNRI; inhibits serotonin > NA reuptake	Serotonergic primarily, dopaminergic at high doses	PD: RCT evidence of benefit for depressive symptoms^123,124^
Duloxetine	Depression, anxiety, neuropathic pain	SNRI; inhibits serotonin > NA reuptake	Serotonergic primarily	PD: RCT failed to show efficacy against pain but improved measures of disease severity (UPDRS) and QoL^125^
Mirtazapine	Depression, insomnia	α2-adrenergic antagonist → ↑ NA and serotonin	5-HT2, 5-HT3 antagonism; sedative	AD: RCT has shown no effect on or agitation with trend towards increased mortality versus placebo.^126^ Further RCT evidence of no benefit on mood symptoms^127^
Bupropion	Depression, smoking cessation	Inhibits NA and DA reuptake	Dopaminergic	AD: No benefit over placebo in treating apathy^128^
Clonidine	Hypertension, ADHD, opioid withdrawal	α2-adrenergic agonist → ↓ NA	No direct serotonin or dopamine effects	AD: Impaired sustained attention in singledose study.^129^ PD: Improved spatial working memory^130^ and impulse control behaviours^131^
Guanfacine	ADHD, hypertension	α2A-adrenergic agonist	Minimal off-target effects	Healthy Ageing: RCT failed to show improvement in executive function^132^
Methylphenidate	ADHD	Inhibits reuptake of NA and dopamine; DA > NA	Dopaminergic primarily	AD: Effective at treating apathy versus placebo.^133-135^ PD: Improved fatigue^136^ and motor function^137,138^, but not cognition^139^
MAO Inhibitors (e.g. selegliline, rasagiline, phenelzine	Parkinsonism, Depression, and anxiety	Inhibits Monoamine oxidase-A/B → ↑ NA, 5-HT, DA	Varying degrees of dopaminergic and serotonergic action	AD: Rasagiline improved FDG uptake in frontal, cingulate and striatal regions.^140^ PD: Rasagiline may slow progression.^141^ PSP: Rasagiline has shown no effect in an RCT^142^
Propranolol	Hypertension, anxiety, tremor	Non-selective β-adrenergic antagonist	Minimal involvement of other neuromodulator systems	AD: Effective at treating disruptive behaviours in the nursing home population^143^ PD: Improves tremor.^144^ Weak evidence that chronic use increases PD risk^145,146^
Dexmedetomidine	Sedation	α2 adrenergic agonist	No serotonin or dopamine effect	Reduced risk of post-operative delirium^147^
Modafinil	Narcolepsy, shift work	Increases NA and dopamine release	Dopaminergic, boosts orexin and histamine	AD: No benefit over placebo in treating apathy.^148^ PD: Mixed reports of benefit against fatigue symptoms^149,150^

NA = noradrenaline; DA = dopamine; 5-HT = serotonin; AD = Alzheimer's disease; PD = Parkinson's disease; and PSP = Progressive supranuclear palsy.

For example, apathy and impulsivity are known to coincide in patients with neurodegenerative illnesses,^153^ and these domains are proposed symptomatic targets. Single-dose research models support restoration of behaviour and surrogate markers of behavioural constructs across a wide spectrum of cognitive pathologies.^152,154-156^ These behavioural changes are accompanied by alterations in activity and connectivity of frontostriatal neurocognitive networks,^155,157^ and the extent of improvement in apathy and impulsivity measures has been associated with locus coeruleus integrity.^69,158^

A meta-analysis of available clinical trials of noradrenergic treatments in Alzheimer's disease confirmed a positive effect on global cognition.^14^ These are a heterogeneous group of trials, with some of the drugs having broader mechanisms of action than purely noradrenergic. However, the primary action of included studies was most likely to be driven by the upregulation or agonism of noradrenaline.

Ways of modulating the noradrenergic system include using selective noradrenaline reuptake inhibitors (e.g. atomoxetine), or less selective serotonin–noradrenaline reuptake inhibitors (e.g. venlafaxine, mirtazapine). Beyond this, we can target the various noradrenergic receptors found throughout the brain. These adrenoreceptors are by convention divided into three groups, namely α1, α2 and β receptors.^159^

The evidence of noradrenergic treatment effects on cognition is not limited to Alzheimer's disease pathology. A randomised control trial has shown that the noradrenergic reuptake inhibitor atomoxetine is helpful for global cognitive performance in Parkinson's disease. This trial did not statistically meet its intended primary endpoint of improving depression, but did improve global cognition.^113^ Atomoxetine improved measures of attention and impulsivity in a randomised controlled trial in mild cognitive impairment due to Parkinson's disease.^114^ A further open-label study has shown atomoxetine to improve executive dysfunction in Parkinson's disease.^115^ Reboxetine, another highly selective noradrenaline reuptake inhibitor, is effective at treating depression in Parkinson's disease.^117^

Methylphenidate has had positive effects on apathy and global cognition in Alzheimer's disease.^133^ Methylphenidate is a reuptake inhibitor of both the dopamine transporter and the noradrenaline transporter. Some of its clinical benefits on apathy can be attributed to the noradrenergic action.^14^ Attempted modulation of apathy using dopaminergic therapies runs the risk of exacerbating impulsivity, which is an undesirable outcome.^160,161^ However, methylphenidate can improve risk-taking behaviour, at least in the behavioural variant of frontotemporal dementia,^162^ which we speculate to be due to a dominant and favourable noradrenergic effect. Dopaminergic agents in isolation do not show this effect.

Idazoxan, an α2 antagonist, has been studied in progressive supranuclear palsy, but the outcome measures focussed primarily on motor features,^163,164^ rather than cognition. It was beneficial towards planning, sustained attention, verbal fluency and episodic memory in patients with frontotemporal dementia.^165^ The selective α2A agonist guanfacine enhances noradrenergic transmission in the prefrontal cortex by selectively activating postsynaptic adrenergic receptors. This results in local inhibition of cAMP signalling, which in turn closes potassium channels that would otherwise reduce network firing. Through this action, guanfacine acts as a noradrenaline mimic.^166^ For symptomatic effect, guanfacine enhances attention in stroke patients^167,168^ and can help agitated patients with dementia,^169^ with limited evidence that it may ameliorate hospital-associated delirium.^170^ Low-dose guanfacine did not improve measures of executive dysfunction (*versus* placebo) in a randomised trial in cognitively healthy elderly adults, although this trial did not selectively recruit those with baseline deficits in executive control.^132^ Guanfacine is currently undergoing a phase 3 trial as an add-on therapy to cholinergic treatment in Alzheimer's disease.^171^

Noradrenergic therapy may also achieve its symptomatic benefits via actions on other neurotransmitter systems important for cognition. For example, noradrenaline directly modulates the effects of other major neurotransmitters like dopamine, γ-aminobutyric acid (GABA) and acetylcholine (ACh).^156,172-174^ The main focus of current symptomatic therapy in Alzheimer's disease is upregulation of the cholinergic system, even though the locus coeruleus is involved earlier and to a proportionately greater extent than the cholinergic nucleus basalis.^175^ So, some of the cognitive benefit seen from noradrenergic therapies could be from downstream upregulation of the cholinergic system. Similarly, the noradrenaline and dopamine systems have a complex relationship, and treatment with a nominally noradrenergic agent may partially exert symptomatic benefit through dopaminergic upregulation in the prefrontal cortex.^176,177^

In summary, the noradrenergic system is a target for symptomatic treatment in patients with apathy or impulsivity.^178,179^ We turn next to the potential of noradrenaline for disease-modifying therapy.

## Noradrenaline treatment as disease-modifying

Key evidence for the hypothesis of noradrenergic treatment as a disease modifier arises from the effect of atomoxetine in mild cognitive impairment, where a randomised control trial showed lower levels of tau and phosphorylated tau in the cerebrospinal fluid of those treated with the drug versus placebo.^112^

There is evidence from epidemiological and cohort studies that the risk of dementia is modified by long-term use of noradrenergic drugs. For example, there is an association between anti-depressant use and subsequent dementia diagnosis,^180^ where such anti-depressants act at least in part via the noradrenergic system. However, there are apparent inconsistencies in the epidemiological associations. For example, β-blocker treatment is associated with a lower risk of a diagnosis of Alzheimer's disease,^181,182^ but a higher risk of Parkinson's disease.^183^ These differences might reflect true differential biological moderation by noradrenergic mechanisms. There are candidate mechanisms by which such noradrenergic treatments might favourably influence the development of neurodegenerative disease or alter disease trajectory. β2 agonist use, as for asthma, has been associated with downregulation of the alpha synuclein gene and diminished Parkinson's risk, so the increased incidence with β-blocker use may be the inverse of this phenomenon. However, such associations need to be interpreted with caution, because of ambiguity in causation, e.g. neuropsychiatric symptoms for which a drug is given may be a prodrome of the dementia itself.

There are multiple mechanisms by which noradrenergic treatments may act to slow the pathogenesis of neurodegenerative disease and dementia. These include the restoration of healthy patterns of LC neuronal firing, modulation of neuroinflammation, changes in the accumulation or clearance of toxic protein aggregates, and indirect effects via modulation of sleep and cardiovascular risk. We first consider the actions of noradrenaline on neurons, microglia, astrocytes, glymphatic and sleep. We then review the evidence for noradrenergic moderation of pathogenesis by direct and indirect routes.

### Restoration of LC neuronal firing patterns

Sustained administration of the noradrenaline reuptake inhibitor reboxetine results in diminished resting tonic firing of noradrenergic neurons in rats.^184^ Likewise, atomoxetine in rats is shown to decrease tonic locus coeruleus activity whilst preserving the evoked response to sensory stimulation—consistent with an increase in the phasic-to-tonic ratio of locus coeruleus firing.^185^ A functional MRI study in healthy controls demonstrated that the dopamine and noradrenaline reuptake inhibitor modafinil reduced baseline tonic firing of the locus coeruleus, with increased task-mediated phasic firing.^89^ That the effect of the drug is opposite to the effect of neurodegeneration on the pattern of tonic-to-phasic firing is consistent with the hypothesis that noradrenergic therapy is restorative of normal physiology.

It is hypothesised that this phasic stimulation of noradrenaline may itself be neuroprotective. While it is difficult to prove the hypothesis that volitional cognitive load is of benefit, observational studies support the idea that mentally stimulating tasks appear to be protective against cognitive impairment.^31,186^ In a rodent model of Alzheimer's disease, phasic stimulation of the locus coeruleus acting as a proxy for novel cognitive load has been shown to be protective against pre-tangle tau.^187^

Despite the depleted noradrenergic cell population in symptomatic stages of Alzheimer's disease, there are paradoxically elevated noradrenaline metabolites in cerebrospinal fluid in early Alzheimer's disease, which may precede significant amyloid deposition.^188^ This might be due to compensatory hyperactivity of the locus coeruleus,^189^ which may potentially improve anti-inflammatory or Aβ clearance mechanisms.^190^ Such early AD-associated LC hyperactivity could be detrimental in the long term, if it exacerbates oxidative stress, free radical cell injury or local noradrenaline metabolite toxicity.^191,192^

### Action at astrocytes

Astrocytes have a regulatory role in CNS homeostasis, promoting chemokine expression in response to local injury.^193^ The action of noradrenaline on astrocyte chemokine production is adaptive. In health, noradrenaline induces the secretion of an array of pro-inflammatory chemokines from astrocytes.^194^ However, in a study with a lipopolysaccharide-induced inflammatory injury, noradrenaline suppressed chemokine expression.^194^ This finding may suggest that in the absence of disease, noradrenaline enables an appropriate inflammatory response to ensure homeostatic balance. However, in the setting of injury, noradrenaline can suppress an exaggerated inflammatory response.

In neurodegenerative disease, where there is both noradrenaline loss and neuronal cell injury, there may be a two-hit phenomenon, with both an exaggerated inflammatory response and perpetuated neuronal injury.

### Action at microglia

In health, most microglia are in an inactive, resting state. With alterations in the local environment, for example, deposition of toxic oligomeric proteins such as β-amyloid, α-synuclein or tau, they become activated.^195^ When exposed to Aβ_1–42_ in vitro, activated microglia rapidly display upregulated transcription of pro-inflammatory genes. This finding is attenuated entirely by the presence of noradrenaline, supporting the theory that noradrenaline is itself anti-inflammatory.^196^ This upregulated inflammatory gene transcription has also been seen in mice with induced locus coeruleus lesions.^196^ One anti-inflammatory mechanism of microglia may be improved phagocytosis. Noradrenaline treatment improves phagocytic response to Aβ_1–42_, while the inverse is seen in mice with locus coeruleus loss. They have less phagocytic response and microglial migration.^197^

Noradrenaline acts through binding to G-coupled α and β adrenoreceptors.^27^ In their resting state, microglia express predominantly the excitatory β2 adrenoreceptor, but following activation, the inhibitory α2 adrenoreceptor predominates.^198^ There are mixed reports of inflammatory response to β2 agonist treatment, potentially reflecting this altered expression in the resting versus activated state.^199,200^ With a healthy, functioning noradrenergic system and no pathologic neurodegeneration, noradrenaline would therefore have an excitatory, pro-inflammatory response with improved phagocytic and chemoattractant responses. In the setting of abnormal protein deposition, however, and resultant local microglial activation, noradrenaline would have an anti-inflammatory effect. As above, noradrenaline depletion as seen in neurodegeneration may drive a pro-inflammatory state.

Neuropathological studies confirm microglial activation in all of the major neurodegenerative diseases,^201^ including Alzheimer's disease,^202^ Parkinson's disease,^203^ progressive supranuclear palsy,^204^ Corticobasal degeneration,^205^ dementia with Lewy bodies,^206^ multiple systems atrophy^207^ and frontotemporal dementia.^208^ Microglia are a key mediator of inflammation within the brain, with their density correlating not only with cross-sectional disease severity but also with prognosis as measured using time to death from PET scans quantifying microglial activation.^209,210^ The significance of microglial activation may vary by disease stage. For example, they may have benefits in stages of disease, to clear β-amyloid^190^ and tau.^211^ At some point, the activated microglia become detrimental,^212^ by synaptic and neuronal phagocytosis and through major seeding of toxic oligomers.^211^ A simplified model of Alzheimer's disease includes microglia-mediated inflammatory response and its promotion of deposition of Aβ deposition and hyper-phosphorylated tau, and subsequent neuro-fibrillary tangle formation, cell toxicity and neurodegeneration.^201^

Noradrenaline is a key down-regulator of microglial activation. Restoring locus coeruleus function through noradrenergic treatments may therefore reduce the microglial activation. In the context of the P301S mouse model of tauopathy, neuroinflammation is accentuated by induced locus coeruleus lesions and resultant noradrenaline deficiency.^213^ Similarly, chemogenetic and pharmacological inhibition of noradrenaline have potentiated a pro-inflammatory phenotype in 5XFAD mice. This potentiation is not seen following selective deletion of beta-adrenergic receptors in microglia.^214^ There are comparable inflammatory changes in Parkinson's disease, with activated microglia promoting the deposition of alpha-synuclein.^215^

The anti-inflammatory benefits of noradrenaline are also protective against the degeneration of dopaminergic neurons.^216^ For example, locus coeruleus lesions in rats potentiate dopaminergic loss. This dopaminergic loss in the rat model is mitigated by the selective noradrenaline reuptake inhibitor, atomoxetine, and potentiated by the α2 antagonist idazoxan.^217^ Specific adrenergic receptors have differing actions on immune response, offering further potential therapeutic options for disease modification. Clenbuterol, a β2 receptor agonist that crosses the blood–brain barrier has shown upregulated IL-1 chemokine release in rat cortex.^218^ Administration of α2 agonists such as dexmedetomidine reduces markers of inflammation in sepsis and systemic illness.^166^

There are other potential anti-inflammatory properties of noradrenaline, with downregulation of inflammatory genes as well as microglia.^219,220^ Targeting neuroinflammation globally, not just specifically within the locus coeruleus, is a plausible goal of noradrenergic therapy.^221^ Recall the risk-modification from anti-depressant agents, many of which have been associated with a reduction in microglial activation, including SNRIs.^222^ Conversely depression is associated with a systemic inflammatory upregulation,^223^ and increased risk of developing dementia.^224^ Some of these effects might arise via activated microglia and cytokines.^206,225,226^ Treatment of depression reduces these systemic markers of inflammation,^227^ although it remains to be seen whether this modifies the risk of dementia.

### Sleep architecture

Noradrenaline is important for wakefulness,^22^ but there is also increasing recognition of the important role it plays in restorative sleep. During non-rapid-eye movement (NREM) sleep, humans and animals are rousable by sensory stimulation, implying some degree of environmental vigilance. This NREM phase of sleep correlates with low levels (but not absent) tonic firing of the locus coeruleus.^228^

Sleep disorders are a common feature across neurodegenerative diseases of ageing. A bidirectional relationship may exist, whereby sleep disorders precede and in turn exacerbate neurodegenerative pathologies.^228^ Noradrenaline dynamics are a key determinant of sleep stages and sleep microarchitecture. Fluctuations in noradrenaline govern the transitions between the rapid-eye-movement (REM) and non-REM sleep cycles,^229^ with sustained low levels of locus coeruleus activity playing a permissive role, allowing for the transition into REM sleep and the maintenance of REM theta.^228^ Noradrenaline fluctuations in non-REM sleep shape sleep microarchitecture, in particular modulating sleep spindles.^230^ Maintenance of NREM sleep and the frequency and timing of sleep spindles are crucial for memory consolidation.^231^ Alterations in spindles are seen in neurodegenerative disease and are associated with poor cognitive performance, offering a potential *in vivo* biomarker of noradrenergic dysfunction.^232^ As locus coeruleus-noradrenergic projections innervate cerebral vasculature and can mediate neurovascular coupling and vasoconstriction,^233^ noradrenaline fluctuations during non-REM are key drivers of the arterial pulsations that facilitate cerebrospinal fluid clearance by the glymphatic system.^234^

### Glymphatics

Although it remains unproven in humans^235^ and is a rapidly progressing field of research^236,237^ the glymphatic system has been proposed to be the major efflux pathway through which toxins and metabolites are excreted from the central nervous system. Disruption of this system may be contributory to dementia pathogenesis.^238^ Microglia and astrocytes, both strongly modulated by the noradrenergic system as above, have important roles to play in the emission of toxins into the glymphatic system through improved phagocytosis and chemokine attraction. MRI sequences have been used to quantify the glymphatic system *in vivo* in humans, but the relevance to neurodegenerative disease and LC in particular remains uncertain.^239^

MR diffusion tensor imaging sequences can quantify the glymphatic system, measured as the diffusion along the perivascular space (ALPS) index. This correlates with locus coeruleus integrity and acts as a significant mediator between locus coeruleus degeneration and cognition in Parkinson's disease.^240^

The glymphatic system is significantly more active during sleep. One theory for this is that noradrenaline may suppress the glymphatic system during wakefulness.^241^ Imaging of glymphatic fluid dynamics supports a positive correlation with slow wave EEG activity in NREM sleep.^242^ There is conflicting evidence on the effect of noradrenaline enhancing or reducing treatment effects on the glymphatic system. Administration of adrenergic antagonists prazosin, atipamezole, and propranolol impaired CSF influx into the glymphatic system of rats.^243^ In contrast, the β-1 agonist dobutamine increases CSF exchange with interstitial fluid in rodents.^244^ Certainly, the glymphatic system is complex, but the noradrenergic system is a key factor in its function, and provides another potential mechanism whereby noradrenaline treatments may alter the course of dementia.

### Amyloid plaques

Animal models of Alzheimer's disease have revealed associations between noradrenaline deficiency and amyloid plaque deposition.^245^ Noradrenergic innervation may be a promoter of amyloid clearance, breakdown of which is felt to be a core step in the pathogenesis of Alzheimer's plaques.^246^ It has been shown in a mouse model that locus coeruleus loss and resultant noradrenaline depletion are associated with a more inflammatory local response to Aβ.^197^ It is postulated that impaired clearance of toxic beta-amyloid (Aβ) plaques and heightened inflammatory response to this accumulating Aβ are key drivers of the neurodegenerative cascade in Alzheimer's disease.

With early locus coeruleus and mild noradrenaline depletion, there are compensatory changes in the adrenoreceptors of the forebrain. Pathological studies support an increase in β_1_ and β_2_ receptors in the hippocampus and frontal cortex, with a reduction in α_1_ receptors in the hippocampus and α_2_ receptors in the prefrontal cortex.^247,248^ Upregulation of the β_2_ receptor may favour Aβ deposition,^249^ and may accentuate the local inflammatory response to Aβ plaques.^250^ This may explain the apparent protective nature of blood–brain barrier-permeable β2 antagonists against Alzheimer's disease.^181^

In a transgenic rat model of amyloid pathology (APP tg), a pharmacologically induced locus coeruleus lesion was associated with subsequent worse cognition at four months post-lesion. This study showed diminished hippocampal cholinergic innervation, reduced neurotrophic gene expression, increased numbers of activated microglia and a pro-inflammatory cytokine profile in the rats with induced locus coeruleus loss.^251^ In the 344–19 transgenic rat model of AD, an anti-noradrenergic neuron toxin-induced lesion of the LC led to increased amyloid and inflammation pathology in forebrain regions versus controls, with early emergence of amyloid angiopathy.^252^

In the APP tg transgenic mouse model, inducing a locus coeruleus lesion was associated with worsened Aβ plaque deposition. More Aβ was seen at 6.5 months post-lesion, with worse cognitive performance, implying that locus coeruleus loss and resultant noradrenaline deficit may accelerate the disease process.^253^ In a similar study of locus coeruleus lesions in mice, there was impaired microglial phagocytosis of amyloid, which recovered with supplementation of the noradrenaline precursor L-DOPS (droxidopa), implying restoration of noradrenaline is a clear potential therapeutic target for improving amyloid clearance.^196^ L-DOPS (droxidopa) has also been shown to be beneficial in 5xFAD transgenic mice, reducing amyloid burden, reducing astrocyte activation and improving memory performance.^254^

Treatment with the α2 antagonist idazoxan reduced hippocampal and cortical Aβ deposition in both APP/PS1 transgenic mice and non-transgenic mice.^255^ Idazoxan appeared neuroprotective in this transgenic model with improved object recognition and spatial memory.

### Tauopathy

Noradrenaline modulates the severity of aggregated tau inclusions that are cytotoxic and synaptotoxic across a range of neurodegenerative disorders. Neurofibrillary tangles can be seen in the locus coeruleus from the age of ten.^2^ Ninety percent of healthy individuals will have some evidence of phosphorylated tau in their locus coeruleus by the age of 30.^2^ The cause of locus coeruleus vulnerability to tau deposition is multifaceted. High metabolic demands and proximity to the fourth ventricle, an area of relatively weak blood–brain barrier, may increase exposure to toxins and oxidative stress.^39,256^

Also, the noradrenaline metabolite, DOPEGAL has been shown to stimulate the aggregation and propagation of tau within the locus coeruleus.^192,256^ In a P301S tau model mouse, reducing levels of DOPEGAL with an MAO-A inhibitor clorgyline increased the number of neurons in the locus coeruleus, and reduced the propagation of tau from brainstem structures to the entorhinal cortex and hippocampus.^192^ A noradrenergic reuptake inhibitor, desipramine, has been shown to reduce the metabolism of noradrenaline. Together, these findings suggest that a further potential benefit of noradrenergic treatment is the reduction in neurotoxic metabolites.^257^

Tau-mediated toxicity is a feature of many clinical dementias, including Alzheimer's disease. Using cerebrospinal fluid tau and phosphorylated tau as *in vivo* biomarkers of Alzheimer's disease pathology, a recent phase II clinical trial with atomoxetine reported significantly reduced levels, *versus* placebo, in mild cognitive impairment due to Alzheimer's disease.^112^

In the P301S transgenic mouse model of tauopathy, a neurotoxin-induced lesion causing locus coeruleus neuronal loss accelerates pathology elsewhere in the brain.^213^ There was greater neuronal loss in the hippocampi of P301S mice with a pharmacologically induced locus coeruleus lesion, and P301S mice displayed worse spatial learning and memory. The P301S mice died earlier than wild-type mice, proportionate to locus coeruleus loss.

Mimicking novel cognitive tasks in a rat model through phasic stimulation of locus coeruleus neurons has been shown to be protective against pre-tangle tau. In the same study, additional tonic stimulation was shown to be harmful and potentiated the progression of pre-tangle tau.^187^ This finding may support restoration of a phasic dominant pattern of locus coeruleus firing as a meaningful treatment target. This phasic dominant firing pattern can be achieved with noradrenergic treatments.^258^

### Cell survival

A further potential protective mechanism of noradrenergic therapies is that they increase brain-derived neurotrophic factor (BDNF) in rats with locus coeruleus lesions.^259^ This has also been seen in humans in one study, elevating BDNF by 24% versus placebo.^112^ BDNF has been implicated as promoting cell survival in neurodegenerative disease.^260^ Low BDNF is seen in Alzheimer's disease^261^ and Parkinson's disease.^262^ Physical exercise is also implicated in endogenous production of BDNF, so the less apathetic patient who now moves more may also derive biological benefit through this pathway.^263^

### Blood–brain barrier

The blood–brain barrier plays a key role in neuronal homeostasis and junk protein clearance.^264^ Dysfunction of this barrier is felt to be both a contributory cause and a resultant consequence of Alzheimer's disease^265,266^ and Parkinson's disease,^267^ and potentially across the neurodegenerative spectrum.^268,269^ Early blood–brain barrier dysfunction may have bidirectionally deleterious effects in neurodegeneration, with impaired protein clearance coupled with disordered immune response.^267^ Rat models of noradrenergic dysfunction have shown structural disorganisation of tight junctions in the blood–brain barrier, and impaired blood–brain barrier permeability in response to systemic hypertension or seizures.^270-272^ And, in the 344-19 transgenic rat model of AD, lesioning the LC leads to disrupted blood–brain barrier permeability.^252^

### Systemic blood pressure

Elevated systemic blood pressure is a well-established risk factor for developing dementia.^273,274^ Serotonin/Noradrenaline Reuptake Inhibitor (SNRI) treatment can elevate blood pressure,^275^ which might mitigate some of the potential therapeutic benefits of noradrenergic therapies outlined above. An adrenergic antagonist (e.g. bisoprolol, nadolol) which does not cross the blood–brain barrier might alleviate this systemic side-effect of SNRI treatment, while maintaining the benefit of supplemental noradrenaline centrally. Treating peripheral side effects has precedent in clinical neurology, e.g. combined carbidopa in dopaminergic therapy in Parkinson's disease. In patients at risk of cardiovascular events, effective blood pressure management may reduce subsequent cognitive decline, so caution regarding iatrogenic hypertension is warranted.^276^

### Via indirect effects

In addition to the direct effect of noradrenaline on pathogenic mechanisms, noradrenergic treatments may affect the long-term risks by moderating behaviours that are themselves detrimental. For example, apathy is a common, early neuropsychiatric feature of many neurodegenerative diseases.^277-279^ It is predictive of cognitive decline and is associated with loss of locus coeruleus integrity.^70,280^ In the absence of any biological disease modification, it is plausible that improving apathy may have a neuroprotective effect.

The exact mechanisms of this effect are not clear. It may be that a treated, less apathetic person is more likely to make better, ‘healthier’ life choices. They may be better nourished, exercise more and be more likely to seek healthcare. Such behaviours are beneficial to our health and quality of life. Apathy has been shown to be correlated with earlier mortality in frontotemporal dementia, progressive supranuclear palsy^281^ and Alzheimer's disease.^282,283^ It is also associated with higher mortality in nursing home residents.^284^ Apathy and behavioural disturbance correlate with earlier care home admission, providing a strong economic argument for targeting these symptoms.^285^ Noradrenergic medication may improve apathy, and in doing so they may improve long-term outcomes.^14,113^

There are extensive neuronal connections between the locus coeruleus and hypothalamus. In Alzheimer's disease, post-mortem studies support locus coeruleus involvement long preceding hypothalamic disease. Isolated noradrenergic degeneration in animal models is sufficient to cause upstream hypothalamic dysfunction with disordered gonadotrophin hormone response, hyporexia, and disordered sleep-wake cycle. These are all common findings in early clinical Alzheimer's disease. It remains to be tested if noradrenergic treatments can favourably alter these hypothalamic functions.^286^

Neurodegeneration and obstructive sleep apnoea have a complex bidirectional relationship,^287^ but noradrenergic treatments have not been assessed alone and in combination with oxybutynin and trazadone in randomised controlled trials in obstructive sleep apnoea to good effect.^288-290^ It remains to be seen if this effect is transferable to cohorts of patients with neurodegeneration-associated obstructive sleep apnoea, and whether treating nocturnal disordered breathing pharmacologically provides cognitive benefit or impacts disease progression.

Another avenue whereby noradrenergic treatments may influence lifestyle and resultant health outcomes in dementia is in the treatment of co-existent autonomic failure, in particular orthostatic hypotension. This is a common and disabling problem in α-synuclein-associated Parkinsonian disorders^291^ and is more common in Alzheimer's disease than healthy controls.^292^ Droxidopa, a noradrenaline pro-drug, has been shown to be effective in a randomised control trial in orthostatic hypotension.^293^ Some of the prognostic benefits of droxidopa may be attributed to a reduction in falls,^294^ enabling a hitherto bedbound patient to mobilise, and reducing risk of sarcopaenia, venous thrombosis and bedsores. Maintaining intracranial perfusion pressure is likely to be beneficial to cognition, and in glymphatic-mediated clearance of further toxic proteins, amongst other homeostatic processes. Accordingly, there may be greater benefit in patients with co-existent cerebral hypoperfusion.

## Areas of contradiction or controversy

Not all effects of noradrenergic treatment are likely to be beneficial. Too much noradrenaline may precipitate symptoms of anxiety and insomnia, or even accelerate pathology.^186^ Here, we consider areas of outstanding controversy or contradictory evidence and highlight areas in need of clarification.

Increasing synaptic activity in a brain that already has dysregulated Aβ clearance may worsen oxidative stress through increased metabolic demand, increasing toxin production further. There may be an as-yet-undetermined threshold after which noradrenergic treatment may actually accelerate pathology.^186^ Further work needs to be done to clarify who is most likely to benefit.

There is conflicting evidence on the impact of noradrenaline on cerebral blood flow, but there is an association between noradrenaline administration and intracerebral vasoconstriction of medium-sized vessels.^295^ This vasoconstriction may have a deleterious effect, through disordered cerebral autoregulation of blood pressure and resultant relative hyperaemia or ischaemia. Co-administration of phentolamine, a non-selective α-receptor antagonist, attenuated this disordered vasoconstriction and autoregulation.^296^ The intracranial vasoconstrictive response to systemic noradrenaline supplementation may be reflective of the degree of preservation of blood-brain-barrier integrity. Certainly, there are important reasons to argue that the relationship of noradrenaline to vascular health may have important effects on symptom burden and progression of dementia.

Noradrenergic therapy has been shown to downregulate GABA in limbic stress circuits in rats.^173^ This downstream reduction in GABA is a further potential mechanism by which SNRI therapy may be helpful in apathy. The cognitive effects of GABA modulation are dose-dependent, with problems arising on either end of this biological spectrum. Supratherapeutic noradrenaline may lead to excessive downregulation of GABA, which has itself been associated with impulsivity in frontotemporal lobar degeneration.^297^

It is difficult to define the direction of effect of sleep disorders in neurodegenerative illnesses, given the known association between brainstem pathology and pathological sleep. But disrupted and diminished rapid-eye-movement (REM) sleep is extremely common in cognitively robust patients taking antidepressants (including noradrenergic agents).^298^ It is recognised that REM sleep is important for consolidation of memory and learning,^299^ but it remains to be shown whether diminished REM sleep alters the course of neurodegenerative illness. Drawing from evidence in REM sleep behavioural disorder (RBD), it seems that the glymphatic system is negatively impacted in this condition.^300^ The glymphatic system is important in the excretion of neurotoxins and metabolites, so its disruption by noradrenergic treatments that affect REM sleep may promote rather than slow pathogenesis.

Noradrenergic neurons are inactive during REM sleep.^301^ They remain active in non-REM sleep, however.^302^ Noradrenergic activity is associated with sleep spindle formation,^228^ and increased LC activity is seen during slow wave activity in NREM following a learning task in rats.^303^ It appears that memory consolidation and restorative sleep are dependent on this increase in noradrenaline in NREM. It remains to be seen if treatment with noradrenergic medication is conducive to an improvement in NREM sleep in the context of neurodegeneration. There is limited sleep-related research on selective noradrenergic therapies, and we cannot draw definitive conclusions, but reboxetine, a noradrenaline reuptake inhibitor, does seem to have less of a negative effect on sleep architecture than primarily serotonergic antidepressant agents.^304^

Taken together, optimal levels of noradrenaline during sleep are required to maintain sleep integrity and to regulate memory consolidation and restorative aspects of sleep. A challenge for noradrenergic drug therapy is to ensure that it does not overwhelm the system and obscure the dynamic fluctuations in noradrenaline levels that are needed for consolidation and restorative sleep. Selective, targeted modulation of subcomponents of the noradrenergic system may be of benefit in promoting healthy sleep. One such option is dexmedetomidine, a selective α2 agonist, which acts to reduce noradrenergic tone by activation of presynaptic α2-autoreceptors. Used in intensive care units following head injuries, it promotes non-REM sleep and enhances glymphatic function.^305^

While this review focusses on treatments that act by increasing noradrenaline levels, there is some evidence to suggest a benefit of noradrenaline-lowering agents in treating aggression and agitation in advanced dementia. Propranolol, a non-selective β-receptor antagonist, has been shown to temporarily reduce symptoms of agitation and aggression in a nursing home cohort with probable or possible Alzheimer's disease.^143^ Prazosin, an α1 antagonist, is potentially helpful in treating agitation in dementia.^306^ A recent randomised clinical trial of prazosin failed to reach statistical significance, but may have been underpowered.^307^ The mechanism by which reducing noradrenaline in advanced disease may reduce symptoms of agitation remains unclear. It may imply that these symptoms of agitation and aggression reflect a relative hyper-vigilant state, which settles with the sedative effect arising from noradrenaline-depleting drugs.

A confounding factor in the interpretation of the noradrenergic system in Parkinson's disease (and to a lesser degree other Parkinsonian syndromes) is the common concomitant prescription of levodopa, which is a precursor of noradrenaline.^52^ Animal studies on the impact of endogenous levodopa on noradrenaline levels in brain tissue have mixed results. In rat models of PD with lesioned dopaminergic systems, endogenous levodopa *reduced* NA levels in striatal neurons^308^ and prefrontal cortex.^309^ Endogenous levodopa does not alter LC electrophysiological firing in experiment-naive rats.^310^

## Conclusion

**Figure 3:**
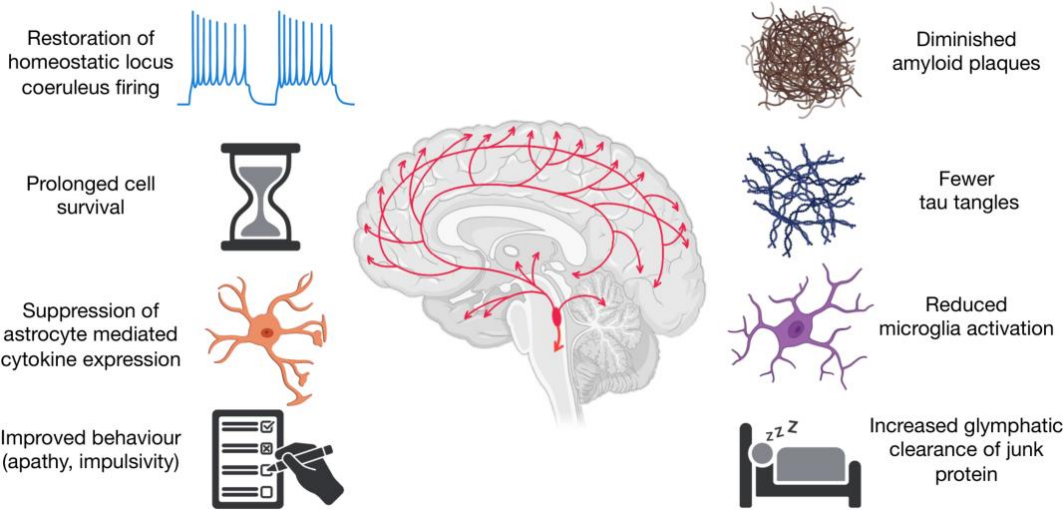
**Mechanisms of potential disease modification: Summary of the multifaceted mechanisms through which noradrenergic treatments can enact a meaningful modifying force on neurodegenerative disease.** Created in BioRender. Durcan, R. (2025) https://BioRender.com/7cwlt6f.

The noradrenergic system is an early area of pathology in many neurodegenerative diseases. Noradrenergic deficits underlie many of the problems experienced by patients and their caregivers, and restoring noradrenergic function has clear potential for symptomatic benefit. There is sufficient evidence now to also test the hypothesis that restoring noradrenergic function is potentially also disease-modifying. The mechanisms by which noradrenergic treatment might exert a disease-modifying effect include reduced neuroinflammation, reduced amyloid and tau deposition, and reductions in deleterious behavioural syndromes like apathy (summarised in Fig. 3). To improve predictors of response to noradrenergic therapies, future research strategies may include PET and MRI based imaging quantification of individual differences in noradrenergic integrity, as well as biomarkers of the noradrenergic target mechanisms. Potential pitfalls of noradrenergic therapy are cardiovascular effects and disruption in sleep architecture, but these are generally mild and should not prevent efforts to test the hypothesis that noradrenergic agents favourably alter the disease course in dementia. Recent clinical trial data are promising,^112^ with both clinical benefit and reductions in molecular biomarkers of disease progression. An advantage of the noradrenergic strategy is that the proposed mechanisms are not unique to a single disease pathology but have transdiagnostic therapeutic potential. It remains to be seen if combination therapy will have a synergistic effect upon more conventionally recognised disease modification strategies, such as therapies directed against amyloid or tau. Clinical trials of noradrenergic treatments for disease modification in dementia are warranted.

## Data Availability

Data sharing is not applicable to this article as no new data were created or analysed in this study.
